# Free Total Rhubarb Anthraquinones Protect Intestinal Injury via Regulation of the Intestinal Immune Response in a Rat Model of Severe Acute Pancreatitis

**DOI:** 10.3389/fphar.2018.00075

**Published:** 2018-02-13

**Authors:** Yuxia Xiong, Li Chen, Ling Fan, Lulu Wang, Yejiang Zhou, Dalian Qin, Qin Sun, Jianming Wu, Shousong Cao

**Affiliations:** ^1^Department of Pharmacology, School of Pharmacy, Southwest Medical University, Luzhou, China; ^2^Department of Pharmacy, Affiliated Hospital of Traditional Chinese Medicine, Southwest Medical University, Luzhou, China; ^3^Department of Gastrointestinal Surgery, Affiliated Hospital of Southwest Medical University, Southwest Medical University, Luzhou, China

**Keywords:** rhubarb, free total rhubarb anthraquinones, sever acute pancreatitis, inflammation, inflammasome, immune function, intestinal mucosal barriery

## Abstract

Intestinal mucosal immune barrier dysfunction plays a key role in the pathogenesis of severe acute pancreatitis (SAP). Rhubarb is a commonly used traditional Chinese medicine as a laxative in China. It markedly protects pancreatic acinar cells from trypsin-induced injury in rats. Free total rhubarb anthraquinones (FTRAs) isolated and extracted from rhubarb display the beneficial effects of antibacteria, anti-inflammation, antivirus, and anticancer. The principal aim of the present study was to investigate the effects of FTRAs on the protection of intestinal injury and modification of the intestinal barrier function through regulation of intestinal immune function in rats with SAP. We established a rat model of SAP by injecting 3.5% sodium taurocholate (STC, 350 mg/kg) into the biliopancreatic duct via retrograde injection and treated the rats with FTRAs (36 or 72 mg/kg) or normal saline (control) immediately and 12 h after STC injection. Then, we evaluated the protective effect of FTRAs on intestinal injury by pathological analysis and determined the levels of endotoxin (ET), interleukin 1β (IL-1β), tumor necrosis factor α (TNF-α), nitric oxide (NO), myeloperoxidase (MPO), capillary permeability, nucleotide-binding oligomerization domain-like receptors 3 (NLRP3), apoptosis-associated speck-like protein containing a CARD domain (ASC), casepase-1, secretary immunoglobulin A (SIgA), regulatory T cells (Tregs), and the ratio of Th1/Th2 in the blood and/or small intestinal tissues or mesenteric lymph node (MLN) cells. Moreover, the chemical profile of FTRAs was analyzed by HPLC-UV chromatogram. The results showed that FTRAs significantly protected intestinal damage and decreased the levels of ET, IL-1β, TNF-α, and NO in the blood and TNF-α, IL-1β, and protein extravasation in the intestinal tissues in SAP rats. Furthermore, FTRAs significantly decreased the expressions of NLRP3, ASC, and caspase-1, the number of Tregs and the ratio of Th1/Th2, while significantly increased the expression of SIgA in the intestinal tissues and/or MLN cells in SAP rats. Our results indicate that FTRAs could protect intestinal injury and improve intestinal mucosal barrier function through regulating immune function of SAP rats. Therefore, FTRAs may have the potential to be developed as the novel agent for the treatment of SAP clinically.

## Introduction

Acute pancreatitis (AP) is a sudden inflammation of the pancreas and one of the most common urgent abdominal diseases. Although most patients with AP are self-limited with low incidence of complications, 10–20% patients may develop to severe acute pancreatitis (SAP) with 30% mortality rate due to secondary infection and organ failure (Thoeni, 2012; Vaz et al., 2013; Xiao et al., 2014). The principal pathological change of SAP is autodigestion from digestive enzymes such as trypsin (Sha et al., 2009). However, the pathogenesis of SAP is complex and the exact mechanism has not been fully clarified. The intestinal tract is the body’s largest immune organ and the impairment of intestinal mucosal barrier function could cause the development of local and systemic septic complications in patients with SAP (Ammori et al., 2002). Intestinal mucosal immune dysfunction induced by bacterial translocation, endotoxemia, and infection is the leading cause of death in patients with SAP (Capurso et al., 2012).

It is generally believed that the intestinal immune response consists of two phases with an initiative hyperinflammatory phase followed by subsequent potential hypoinflammatory phase in SAP (Mylona et al., 2011; Gunjaca et al., 2012). The initial hyperinflammatory phase in SAP is characterized by activation of innate immune cells such as monocytes, macrophages and other immune cells to release a large amount of proinflammatory cytokines such as TNF-α, IL-1β, IL-1, IL-6, IL-8, IFN-γ, platelet activation factor, and the chemokines (Makhija and Kingsnorth, 2002; Mylona et al., 2011; Gunjaca et al., 2012). The hypoinflammatory or immunosuppressive phase in SAP occurred in the later stage is characterized as “counter anti-inflammatory response” by induction of IL-10 and IL-1 receptor antagonist and drift of T-helper cells (Makhija and Kingsnorth, 2002; Mylona et al., 2011). Study has shown that the intestinal barrier plays an important role in SAP (Tian et al., 2013). The mucosa-associated lymphoid tissues in intestine constitute the intestinal immune barrier to effectively prevent the invasion of bacteria and endotoxin (ET, lipopolysaccharide) from intestine into the blood (ZhaXi et al., 2014). However, bacteria and ET may pass through the intestine into the sterile abdominal cavity (peritoneum) to cause serious complications when the intestinal barrier function is weakened or damaged (Vaishnavi, 2013). Therefore, it may be an effective strategy for the prevention and treatment of SAP through regulation of intestinal immune function and restoration of normal intestinal mucosal barrier.

Rhubarb is a commonly used traditional Chinese medicine as a laxative in China. Studies from clinical trials have shown that rhubarb has beneficial effect on patients with SAP for significant improvement of the gastrointestinal function and inhibition of systemic inflammation (Wan et al., 2014; Zhou et al., 2016). Rhubarb markedly protects pancreatic acinar cells from trypsin-induced injury by inhibiting the synthesis, secretion and release of trypsin in rats (Zhu et al., 2014). Furthermore, rhubarb reduces the permeability of the capillaries, inhibits the releases of inflammatory cytokines and oxygen free radical, promotes intestinal goblet cell hyperplasia, and improves microcirculation of intestinal mucosa (Neyrinck et al., 2017). However, rhubarb has some disadvantages such as containing too many ingredients and great variation in efficacy. In addition, a large dose of traditional decoction is needed for the treatment of SAP and it may aggravate the gastrointestinal burden of patients to cause more complications and the mechanistic action is still unclear. Thus, these shortcomings limit its clinical applications for the treatment of SAP. On the contrary, free total rhubarb anthraquinones (FTRAs) isolated and extracted from rhubarb, have the advantages over rhubarb including relatively small number of ingredients, easier quality control, smaller dose for disease treatment and better clinical applications (Zhang et al., 2013, 2015).

Although the effect of rhubarb on modulation of intestinal immune functions has been demonstrated (Yang et al., 2012), the effect of FTRAs on the intestinal immune function and underlying mechanisms remain unclear in the treatment of SAP. Our previous studies have demonstrated that rhubarb and FTRAs have equivalent effect to ameliorate the injury of intestine, liver and kidney by reducing inflammatory cascades mediated by cytokines and systemic inflammatory response in rats with SAP (Liu et al., 2015; Wang et al., 2015; Chen et al., 2016). We hypothesized that FTRAs may display significant effect on organ protection via regulating the intestinal immune response at early stage in the treatment of SAP. In order to overcome the shortcomings and improve the therapeutic effect of rhubarb, we aimed to provide the scientific rationale for the development of FTRAs as a more efficacious novel agent for the treatment of SAP clinically. Therefore, we selected FTRAs for the present studies.

In the present studies, to rationally design the experiments, we first investigated the effects of FTRAs on intestinal injury protection and determination of ET, TNF-α, IL-1β, NO, and MPO in blood and/or in small intestinal tissues in SAP rats. We also studied the possible associated mechanisms with the therapeutic effects of FTRAs by investigating protein extravasation and the expressions of NLRP3, ASC and caspase-1 in the small intestinal tissues and mesenteric lymph node (MLN) cells in SAP rats to evaluate the non-specific (innate) immune response of intestine in SAP rats. Furthermore, we also investigated the effects of FTRAs on the expressions of SIgA, Th cells and regulatory T cells (Tregs) in the small intestines or MLN cells to assess the specific (adaptive) immune response of intestine in SAP rats. In addition, we also studied the chemical profile of FTRAs for future development of active purified compound. The results show that FTRAs could markedly protect intestinal damage induced by SAP and reduce the levels of inflammatory factors TNF-α and IL-1β, ET, and NO in the blood and/or intestine. FTRAs also regulate mucosal function through the downregulation of the expression of NLRP3 inflammasome, increase of the expression of SIgA, decreased the number of Tregs, and restoration of the balance of Th1/Th2. Therefore, the results may provide a scientific rationale for the development of FTRAs as a novel agent to overcome the shortcomings of rhubarb for the treatment of SAP clinically.

## Materials and Methods

### Materials and Regents

Free total rhubarb anthraquinones were purchased from the Research Institute (Huaian, Jiangsu, China, batch number: 20130211) and dissolved in normal saline (NS). Sodium taurocholate (STC) was purchased from Sigma–Aldrich Co. (St. Louis, MO, United States). Rat TNF-α and IL-1β ELISA Kits were purchased from United States Systems R&D (Long Beach, CA, United States). NO Kit was purchased from Beyotime Biotechnology Research Institute (Shanghai, China). MPO kit was purchased from Nanjing Jiancheng Biological Engineering Institute (Nanjing, Jiangsu, China). Fixative solution, permeabilization wash buffer, Foxp3 fixative permeabilization wash solution, monensin, APC anti-rats CD4, FITC anti-rats IFN-γ, PE anti-rat IL-4, PE anti-rat CD25, Alexa fluor 488 anti-rat Foxp3 and goat anti-rat SIgA were purchased from Biolegend Company (San Diego, CA, United States). Rabbit anti-rat NLRP3, goat anti-rat ASC, mouse anti-rat caspase-1 were purchased from Santa Company (Santa Cruz, CA, United States). Triton, bovine serum albumin (BSA) DAB color liquid, goat super sensitive two-step reagent Kit, mouse one-step kit, TRITC labeled rabbit ant- goat secondary antibodies, fluorescein (FITC) labeled sheep anti-rabbit secondary antibodies were purchased from ZSGB Biological Company (Beijing, China).

### Experimental Animals

Six week-old specific pathogen free (SPF) grade male Sprague Dawley (SD) rats (body weight ∼200 g) were purchased from the Experimental Animal Center of Southwest Medical University (Luzhou, Sichuan, China, Certificate No. SCXK2013-24). The rats were housed in plastic cages up to four rats per cage with free access to water and food at a constant room temperature (∼22°C ± 1°C) under a 12 h light/12 h dark cycle. All animal experiments were performed strictly in accordance with University guidelines and were approved by the Committee on Use and Care of Animals of Southwest Medical University (Luzhou, Sichuan, China).

### ASP Model Establishment, Experimental Design, and Drug Treatment

Rats were fasting for 12 h before injection of 3.5% STC (350 mg/kg) or NS (1 ml/kg, as control) into the biliopancreatic duct via retrograde injection to establish the ASP model. The rats were randomly divided into four groups with 14 rats for each group, control group: normal rats treated with NS; SAP group: SAP rats treated with NS; FTRAs-1 group: SAP rats treated with 36 mg/kg FTRAs; and FTRAs-2 group: SAP rats treated with 72 mg/kg FTRAs. The dose selection for FTRAs was first estimated from the dose of rhubarb clinical used for the treatment of patients with SAP and converted from the calculation of the body surface area of rat/human and the extraction ratio of FTRAs from Rhubarb. Then, we performed several pre-experiments with different doses and determined FTRAs at 72 mg/kg or lower dose to be the appropriate dose for the study. Treatments were initiated immediately and 12 h after STC injection by intragastric administration. Eight rats were anesthetized by intraperitoneal (IP) injection of 50 mg/kg of 2.0% pentobarbital sodium 24 h after treatment. The blood was drawn from the abdominal aorta of rats. Plasma and serum were prepared for determination of ET, TNF-α, IL-1β, and NO. Rats were sacrificed after blood collection, and the small intestinal tissues and MLN cells were collected. Parts of small intestine and MLN were stored at -80°C, for determination of TNF-α, IL-1β, and MPO or single cell suspension, and the rests were fixed in 10% buffered formaldehyde for histopathological analysis and examination of NLRP3, ASC, casepase-1 and SIgA. The remaining six rats were used for the evaluation of intestinal permeability.

### Histopathological Analysis of Small Intestines (Ilea) of Rats

The ilea tissues were weighed and fixed in 10% poly formaldehyde for 4 h, then wished with water for 24 h, and embedded in conventional paraffin for microtome sections with hematoxylin and eosin (H&E) stain for pathological study under light microscope (200×) (Olympus Corp., model CX31, Shinjuku-Ku, Tokyo, Japan) and immune index detection. There were eight rats used for each group.

### Determination of ET, MPO and Inflammatory Factors in the Blood and Small Intestinal Tissues in SAP Rats

Endotoxin was determined by azo matrix chromogenic limulus test, NO by Griess, TNF-α and IL-1β by ELISA, and MPO by MPO assay kit followed the manufactures’ instruction. There were eight rats used for each group.

### Detection of the Capillary Permeability in Intestinal Tissue in SAP Rats

Rats were injected with Evans blue (30 mg/kg) in femoral vein 30 min before being sacrificed, and a section of ileum (∼200 mg) was taken and placed into a tube (3 ml/100 mg) and added 3 ml formamide per 100 mg ileum. After 24 h reaction, 1 ml leachate was taken out and measured at the absorbance 620 nm by a spectrophotometer (Molecular Devices Corporation, Sunnyvale, CA, United States). EB standard curve was established by dilution method (0.15625–80 μg/ml) to calculate the EB content as described previously (Cheng et al., 2016). Rests of the ileum were weighed and placed in an oven at 80°C bake for 72 h for determination of the ratio of wet/dry weight as described previously (Nakagawa et al., 2009). There were six rats used for each group.

### Detection of the Expressions of NLRP3 and ASC by IF Assay

The paraffin sections of small intestine and MLN cells were baked in oven at 65°C for 2 h, and dewaxed with xylene I, xylene II, and xylene III for 10 min each following soaking in 100, 95, and 80% ethanol in proper order for 5 min, respectively, then placed in 70% ethanol and distilled water for another 2 min for gradient hydration. The sections were washed with PBS for three times with 5 min each time after hydration, added 0.2 ml of 3% H_2_O_2_ and incubated for 10 min to block endogenous peroxidase, then washed with PBS for three times again with 5 min each time. Sections were placed into pH 6 citric acid repair solution and boiled at 95°C for 20 min, and then naturally cooling to room temperature to repair the antigen. The sections were washed with PBS for three times with 5 min each time, added 0.3% TritonX-100 for 10 min and rinsed with PBS for three times with 5 min each time, then added 5% FBS solution sealing for 30 min. Then, the primary antibody (1:50) was added into the sections and left in the refrigerator at 4°C for overnight. The next day, the sections were warmed 1 h and washed with PBS for three times with 5 min each time. Fluorescent second antibody (1:100) was added in the sections and placed at 37°C in dark place to avoid light for 30 min. Then, the sections were washed with PBS for three times with 5 min each time, and taken photographs under an inverted phase contrast fluorescent microscope (AMG EVOS, Thermo Fisher Scientific Inc., Waltham, MA, United States) as described previously (Guo et al., 2014). There were eight rats used for each group.

### Detection of the Expression of Caspase-1 by IHC Analysis

The sections were dewaxed for graded ethanol hydration, antigen retrieval, endogenous peroxidase blocking as the same processes as IF assay described above. Then, the primary antibody (1:50) was added in the sections after blocking endogenous peroxidase and placed in a refrigerator at 4°C for overnight. The next day, the sections were warmed for 1 h and then washed with PBS for three times with 5 min each time. The second antibody (1:500) was added into the section and placed at 37°C for 20 min, then washed with PBS for three times with 5 min each time. DAB solution was added into the sections for 5 min and rinsing with distilled water for three times with 5 min each time for color display. The sections were stained with H&E and washed with water for 5 min for nuclear staining and observed under a microscope (Olympus Corp., model CX31, Shinjuku-Ku, Tokyo, Japan). The sections were stained with H&E for a second time if the stain was oligochromasia or differentiated with hydrochloride acid and stained with H&E again if the stain was hyperchromasia. Then, the sections were placed in 70, 80, 95, and 100% ethanol, respectively, with 5 min each for gradient dehydration. Finally, the sections were transparent with xylene for 10 min and coated with neutral balata for IHC analysis. There were eight rats used for each group.

### Determination of the Ratio of Th1/Th2 by Flow Cytometry

Mesenteric lymph node cells (1 × 10^6^, 1 ml) were placed in a well of sterile 6-well plate with per-cold (4°C) PBS, and cultured with 2 μL (1:500) monensin for 5 h in an incubator with 5% CO_2_, at 37°C. Then the cells were collected and centrifuged at 350 × *g* for 5 min. The supernatants were discarded and the cells were resuspended in 2 ml PBS, transferred into an EP tube, centrifuged at 350 × *g* for 5 min and discarded the supernatants, then 100 μL cell resuspension was transferred into another EP tube. CD4 antibody (5 μL, 1:20) was added and incubated at room temperature for 15 min followed by adding 1 ml PBS, centrifuged at 350 × *g* for 5 min and discarded the supernatants. The cells were fixed in stationary liquid (0.5 ml) and incubated for 20 min at the room temperature after mixture. The cells were resuspended in PBS (500 μL), centrifuged at 350 × *g* for 5 min and discarded the supernatants, then added another 500 μL PBS to resuspend the cells and placed in a refrigerator at 4°C for overnight. The next day, the cell resuspensions were centrifuged at 350 × *g* for 5 min and discarded the supernatants. Permeabilization wash buffer (100 μL) was added for cell suspension, centrifuged at 350 × *g* for 5 min and discarded the supernatants. Then, permeabilization wash buffer (100 μL) was added into the cells again to make resuspension followed by added 5 μL IL-4 (1:20) or 20 μL IFN-γ (1:5) and incubated at room temperature for 20 min, washing with PBS for two times, centrifuged at 350 × *g* for 5 min and discarded the supernatants. Finally, PBS (0.3 ml) was added to make suspension to determine the ratio of Th1/Th2 by flow cytometry (FACSCalibur, BD Biosciences, San Diego, CA, United States) as described previously (Takahashi et al., 2017). There were eight rats used for each group.

### Determination of Tregs by Flow Cytometry

CD4 and CD25 antibodies (1:20, 5 μL each) were added into 100 μL (1 × 10^6^ cells) MLN cell suspensions and incubated at room temperature for 20 min, then washing with 2 ml PBS, centrifuged at 250 × *g* for 5 min and discarded the supernatants. Permeabilization wash buffer (1 ml) was added into the cell resuspensions, centrifuged at 250 × *g* for 5 min and discarded the supernatants. then, adding permeabilization wash buffer (100 μL) and FOXP3 antibody (1:20, 5 μL) incubated at room temperature for 30 min. PBS (200 μL) was added into the cell supernatants and centrifuged at 250 × *g* for 5 min, and discarded the supernatants. Finally, PBS (300 μL) was added to make cell suspension for flow cytometry (FACSCalibur, BD Biosciences, San Diego, CA, United States) analysis of Tregs as described previously (Fontenot et al., 2003). There were eight rats used for each group.

### Analysis of the Chemical Profile of FTRAs

The chemical profile of FTRAs was performed at 254 nm wavelength and each UV spectrum peak was compared to the standard compounds by an Agilent 1260 HPLC system (Agilent Technologies, Santa Clara, CA, United States). Chromatographic analysis was performed at 25°C with an Inert Sustain C18 column and water-phosphoric acid (100:0.1, v/v) and methanol was used as the mobile phases A and B, respectively. The mobile phase was delivered at a rate of 0.8 ml/min with 1 μl injection volume. The gradient separation process was as follows: 15–15% B at 0–2 min, 15–80% B at 2–5 min, 80–80% B at 5–7 min, 80–15% B at 7–7.5 min. The data were analyzed by LabSolutions software version 5.75 (Shimadzu Corp., Nakagyo-Ku, Kyoto, Japan).

### Statistical Analysis

All data were expressed as mean ± standard deviation (SD). Statistical analyses were carried out using SPSS 18.0 statistical software (SPSS Inc., Chicago, IL, United States). LSD (L) test was used for homogeneity of variance between the groups compared with single factor, while Dunnett’s T3 test and Pearson correlation were used to evaluate the correlation between different groups. A difference at *P* < 0.05 was considered to be statistically significant (marked as ^∗^). The higher significance level was set at *P* < 0.01 (marked as ^∗∗^).

## Results

### FTRAs Protects Intestinal Damage and Reduces the Levels of ET, Inflammatory Factors TNF-α and IL-1β, and NO in SAP Rats

**FIGURE 1:**
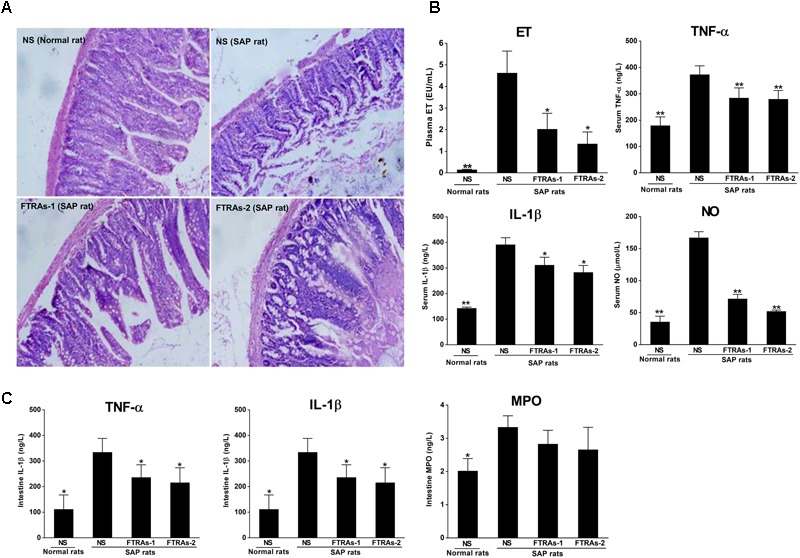
Effects of free total rhubarb anthraquinones (FTRAs) on intestinal injury protection and the expressions of ET, IL-1β, TNF-α, NO, and MPO in the blood and/or intestinal tissues in SAP rats. **(A)** NS (Normal rat): Normal rats treated with 1 ml/kg normal saline (NS) as control; NS (SAP rats): SAP rats treated with 1 ml/kg NS; FTRAs-1 (SAP rat): SAP rats treated with FTRAs 36 mg/kg; and FTRAs-2 (SAP rat): SAP rats treated with FTRAs 72 mg/kg. **(B)** The levels of ET, TNF-α, IL-1β, and NO in the blood in rats. **(C)** The levels of IL-1β, TNF-α, and MPO in small intestinal tissues in rats. Treatment was initiated immediately and 12 h after SAP was induced by STC. The intestinal sections were stained by H&E stain and observed under a microscopy (200× magnification). The data were presented as mean ± SD. There were eight rats in each group (*n* = 8). ^∗^*p* < 0.05, ^∗∗^*p* < 0.01 vs. SAP rats treated with NS by LSD (L) test, Dunnett’s T3 test, and Pearson correlation analysis.

First, we investigated the effects of FTRAs on intestinal injury and the levels of ET, TNF-α, IL-1β, and NO in blood and TNF-α, IL-1β, and MPO in small intestinal tissues in SAP rats treated with FTRAs or NS 24 h after STC injection. As showed in **Figure 1A**, SAP rats induced by STC showed severe small intestinal damage with intestinal wall congestion, edema, hemorrhage, and inflammatory cell infiltration, lower villi height with partial loss and defect, mucosal epithelial cells degeneration and necrosis, separated natural layer and expanded mucosal and submucosal layers. However, treatment of FTRAs with both doses (36 and 72 mg/kg) markedly ameliorated intestinal damage. Moreover, the levels of ET, TNF-α, IL-1β, and NO in the blood and TNF-α, IL-1β, and MPO in the small intestinal tissues were significantly increased (*p* < 0.05 or *p* < 0.01) in SAP rats compared to that of the control rats. However, FTRAs (36 and 72 mg/kg) significantly inhibited (*p* < 0.05 or *p* < 0.01) the elevated levels of ET, TNF-α, IL-1β, and NO induced by SAP in a dose dependent manner in the blood compared to that of SAP rats treated with NS (**Figure 1B**). Similarly, FTRAs at both doses also significantly inhibited the elevated levels of TNF-α and IL-1β (*p* < 0.05) with reduced MPO but without statistical significance (*p* > 0.05) in the small intestine in SAP rats compared to that of NS treatment (**Figure 1C**). The results indicate that FTRAs are effective in protection of intestinal injury and inhibition of the levels of ET, inflammatory factors TNF-α and IL-1β and NO in SAP rats.

### FTRAs Attenuated Intestinal Permeability in SAP Rats

**FIGURE 2:**
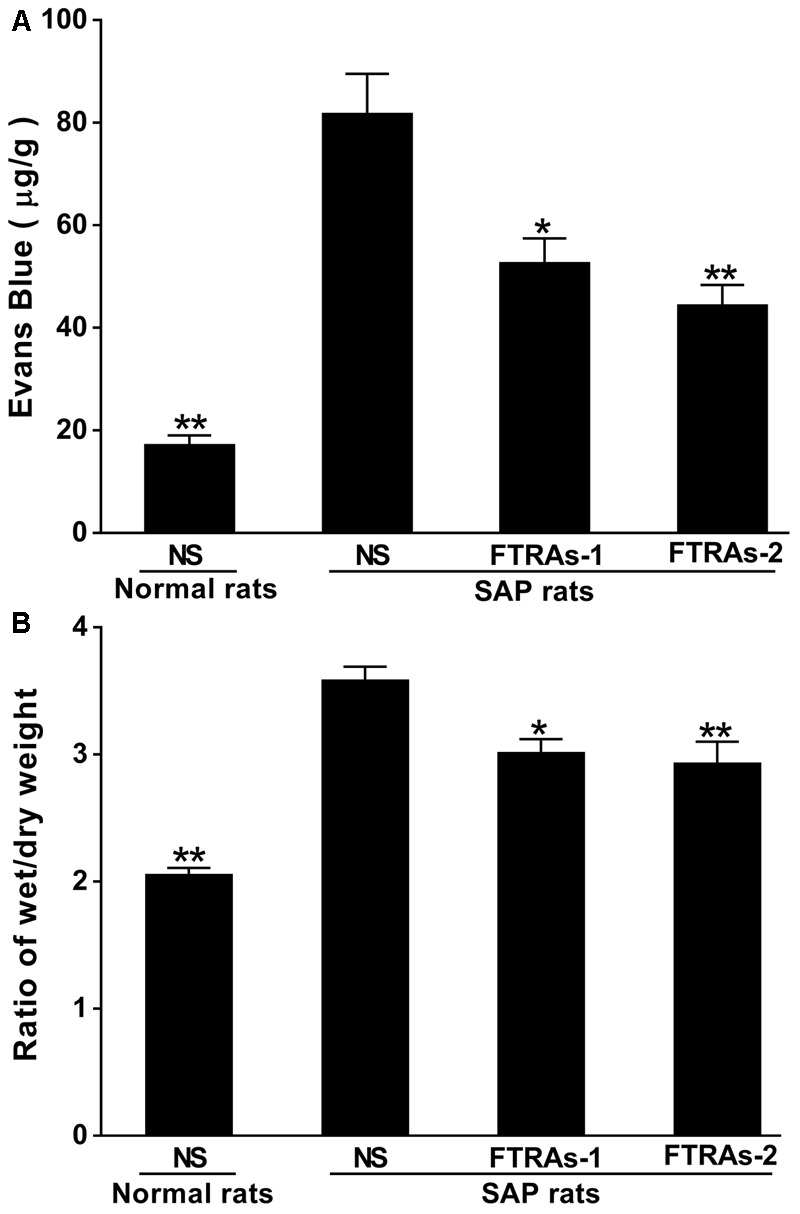
Effect of FTRAs on intestinal permeability by Evans blue (EB) stain **(A)** and the ratio of wet/dry weight of intestinal tissues **(B)** in SAP rats. NS (Normal rat): Normal rats treated with 1 ml/kg NS as control; NS (SAP rats): SAP rats treated with 1 ml/kg NS; FTRAs-1 (SAP rat): SAP rats treated with FTRAs 36 mg/kg; and FTRAs-2 (SAP rat): SAP rats treated with FTRAs 72 mg/kg. Treatment was initiated immediately and 12 h after SAP was induced by STC. The data were presented as means ± SD. There were six rats in each group (*n* = 6). ^∗^*p* < 0.05, ^∗∗^*p* < 0.01 vs. SAP rats treated with NS by LSD (L) test, Dunnett’s T3 test, and Pearson correlation analysis.

Evans blue (EB) is closely attached to albumin, so it is used as a common indicator for protein extravasation in inflammatory tissue injury (Cheng et al., 2016). Therefore, we evaluated the intestinal microvascular permeability by EB staining and the ratio of wet-dry weight of intestinal tissues. As showed in **Figure 2A**, higher dye extravasation was observed in the SAP rats compared to that of normal rats (*p* < 0.01). However, treatment of FTRAs (36 and 72 mg/kg) significantly (*p* < 0.05 or *p* < 0.01) attenuated the extravasation in a dose dependent manner evidenced by blocking the leakage of Evans blue in SAP rats. Furthermore, the ratio of wet-dry weight of intestinal tissues was also significant increased (*p* < 0.01) in SAP rats compared to that of normal rats. However, FTRAs markedly decreased (*p* < 0.05 or *p* < 0.01) the ratio of wet-dry weight of intestinal tissues in a dose dependent manner in SAP rats compared to that of NS treatment (**Figure 2B**). The results indicate that FTRAs could attenuate elevated intestinal permeability to prevent bacteria and ET into the blood in SAP rats. Because FTRAs at the dose of 72 mg/kg has better effects on protection of intestine damage, inhibition of ET, inflammatory factors TNF-α and IL-1β, and NO, and reduction of intestinal permeability induced by SAP than that of 36 mg/kg. Therefore, FTRAs 72 mg/kg was chosen for the follow-up mechanistic studies.

### Effects of FTRAs on the Expressions of NLRP3, ASC and Caspase-1 in the Small Intestinal and MLNs Cells in SAP Rats

**FIGURE 3:**
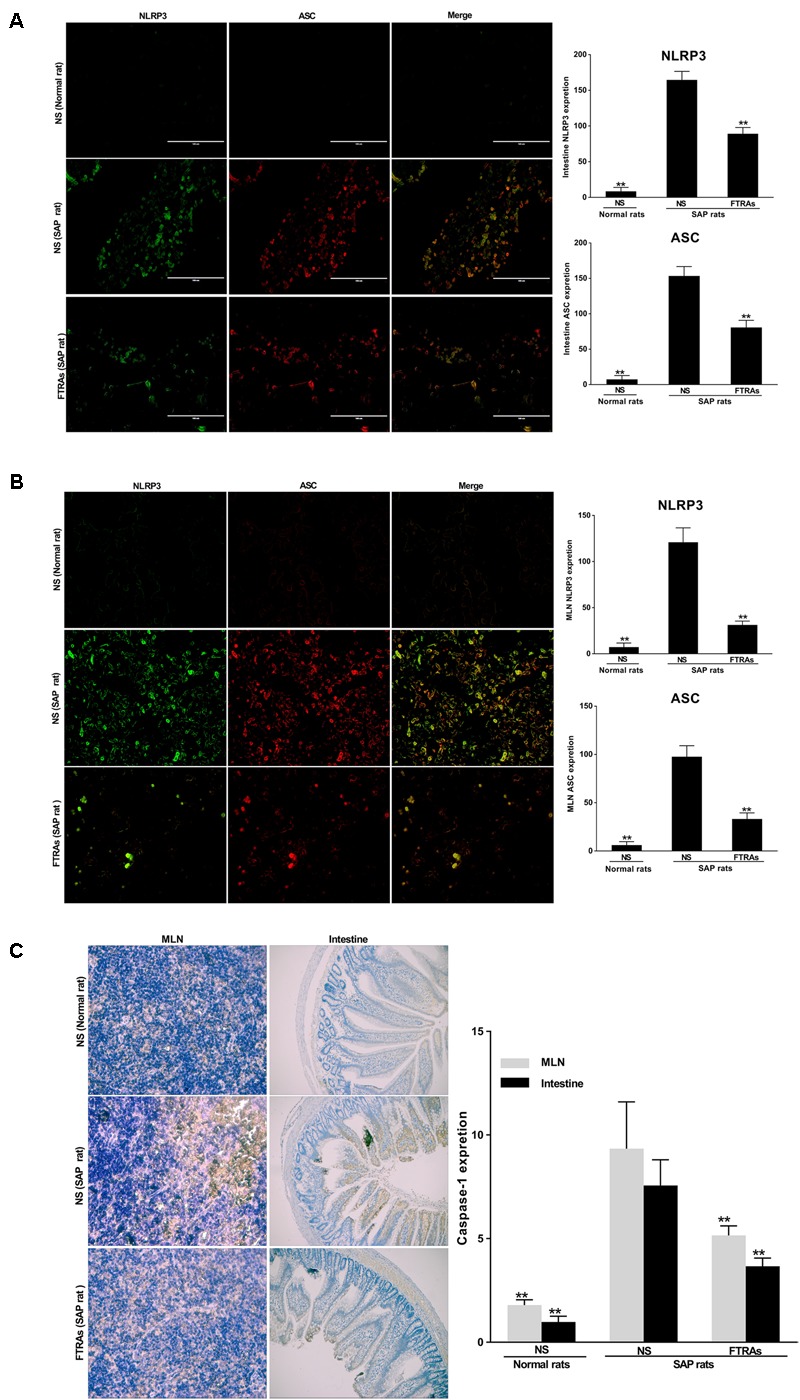
Effects of FTRAs on the expressions of NLRP3, ASC, and caspase-1 in the small intestinal tissues and MLN cells in rats. **(A)** The representative images of the uptake of NLRP3 and ASC in the small intestinal tissues of rats and summary results of bar graphs for each group. **(B)** The representative images of the uptake of NLRP3 and ASC in the MLNs cells of rats and summary results of bar graphs for each group. NLRP3 and ASC were determined by confocal laser scanning microscopy (400× magnification), positive expression of NLRP3 presented as green fluorescence, while positive expression of ASC presented as red fluorescence. The counts of positive expressions of ASC and NLRP3, the fluorescence expressions were counted at least 10 fields for each slide. **(C)** The expressions of caspase-1 in the small intestinal tissues and MLNs cell of rats by immunochemical (IHC) analysis. NS (Normal rat): Normal rats were treated with 1 ml/kg NS as control; NS (SAP rats): SAP rats were treated with 1 ml/kg NS; and FTRAs (SAP rat): SAP rats were treated with 72 mg/kg FTRAs. Treatment was initiated immediately and 12 h after SAP was induced by STC. There were eight rats used for each experimental group (*n* = 8) and expressed as mean ± SD. ^∗^*p* < 0.05, ^∗∗^*p* < 0.01 vs. SAP rats treated with NS by LSD (L) test, Dunnett’s T3 test and Pearson correlation analysis.

To evaluate the effect of FTRAs on the non-specific immune (innate immunity) response of the intestinal tract in the SAP rats, first, we investigated the effects of FTRAs on the expressions of NLRP3 and ASC in the sections of small intestines and MLN cells by IF. The results demonstrate that a great number of positive expressions of NLRP3 and ASC in the sections of small intestines (**Figure 3A**) and MLN cells (**Figure 3B**) in the SAP rats treated with NS compared to that of the control rats treated with NS (*p* < 0.01). NLRP3 expression was mainly localized in the cytoplasm, while ASC expression was uniformly localized (**Figures 3A,B**). Interestingly, FTRAs (72 mg/kg) significantly decreased (*p* < 0.01) the expressions of NLRP3 and ASC in the small intestines and MLN cells compared to that of NS treatment in SAP rats (**Figures 3A,B**). Next, we studied the effect of FTRAs on the expression of caspase-1 in the sections of small intestines and MLN cells by IHC (**Figure 3C**). The results show that the expression of caspase-1 was also markedly elevated (*p* < 0.01) in the small intestines and MLN cells in SAP rats compared to that of normal rats (**Figure 3C**). However, FTRAs (72 mg/kg) partially but significantly decreased (*p* < 0.01) the expression of caspase-1 in small intestines and MLN cells in SAP rats (**Figure 3C**). The data indicate that the expressions of NLRP3, ASC and caspase-1 are markedly increased and FTRAs are effective in decreasing their expressions in SAP rats.

### Effects of FTRAs on the Levels of SIgA, Th Cells and Tregs in the Small Intestines and MLN Cells in SAP Rats

**FIGURE 4:**
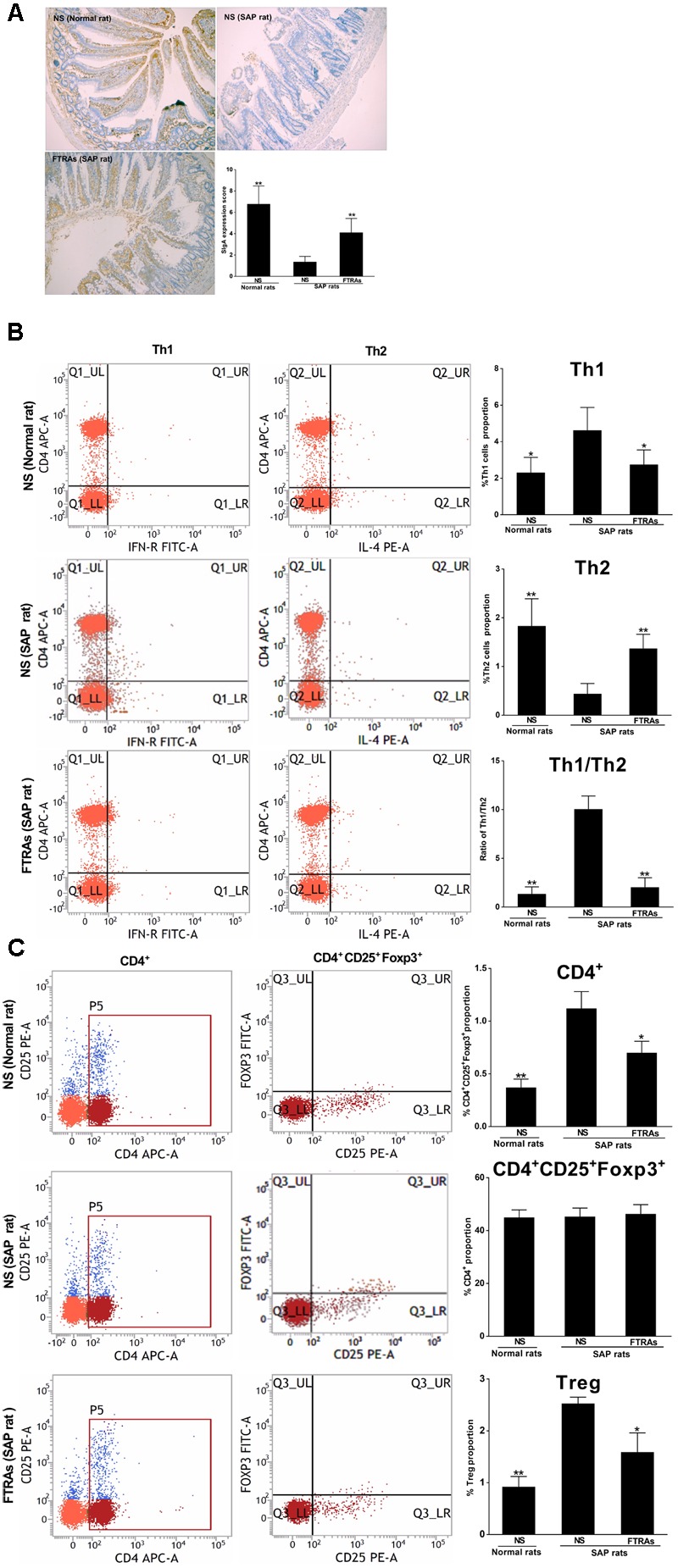
Effects of FTRAs on the levels of SIgA in small intestinal tissues by IHC analysis **(A)**, Th1 cells, Th2 cell, and Th1/Th2 ratio **(B)** and CD4+, CD4 + CD25 + Foxp3, and Tregs in MLN cells **(C)** by flow cytometry in rats. NS (Normal rat): Normal rats were treated with 1 ml/kg NS as control; NS (SAP rats): SAP rats were treated with 1 mg/kg NS; and FTRAs (SAP rat): SAP rats were treated with 72 mg/kg FTRAs. Treatment was initiated immediately and 12 h after SAP was induced by STC. Eight rats were used for each experimental group (*n* = 8) and expressed as the mean ± SD. ^∗^*p* < 0.05, ^∗∗^*p* < 0.01 vs. SAP rats treated with NS LSD (L) test, Dunnett’s T3 test and Pearson correlation analysis.

SIgA, Th cells and Tregs play a key role in the specific immune (adaptive immune) response. Therefore, we evaluated their role in the pathogenesis of SAP and the effect of FTRAs in SAP rats. We first studied the effect of FTRAs on SIgA in intestines and MLN cells, a key player in humeral immunity and the first line of intestinal mucosal defense. The results showed that the level of SIgA was markedly decreased (*p* < 0.01) in the small intestine in SAP rats compared to that of normal rats. However, the level of SIgA was significantly up-regulated (*p* < 0.01) in SAP rats treated with FTRAs (72 mg/kg) compared to that of SAP rats treated with NS (**Figure 4A**). Th cells are the central cells of the body’s specific immune response. Next, we studied the effect of FTRAs on Th cells in SAP rats. The expressions of CD4^+^ IFN-γ^+^ and CD4^+^ IL-4^+^ present the populations of Th1 and Th2 cells, and the ratio of Th1/Th2 represents their balance. The results showed that expression of Th1 cells (*p* < 0.05) and the ratio of Th1/Th2 (*p* < 0.01) were significantly increased but the expression of Th2 (*p* < 0.01) was markedly decreased in SAP rats compared to that of normal rats, indicating that Th1/Th2 is seriously imbalance in SAP rats. Interestingly, FTRAs significantly decreased the expression of Th1 (*p* < 0.05) and increased the expression of Th2 (*p* < 0.01) compared to NS treatment in SAP rats, therefore, restored the balance of Th1/Th2 (*p* < 0.01) to near normal level as control (normal rats treated with NS) (**Figure 4B**). Tregs have an essential role in maintaining the balance between immune activation and tolerance. Finally, we studied the effect of FTRAs on Tregs in SAP rats. The transcription factor CD4^+^Foxp3 of Tregs is the most specific marker for Tregs. The results showed that the positive cells of CD4^+^CD25^+^Foxp3^+^ and population of Tregs were significantly increased (*p* < 0.01) but the population of selected CD4^+^ T cells unchanged in SAP rats compared to that of normal rats. However, FTRAs significantly decreased (*p* < 0.01) the elevated positive cells of CD4^+^CD25^+^Foxp3^+^ and population of Tregs compared to that of NS treatment in SAP rats (**Figure 4C**). The data indicate that SIgA is significantly decreased but the population of Tregs is significantly increased and Th1/Th2 is seriously imbalance in SAP rats. However, FTRAs are effective in modulation of the specific immune response of the intestinal tract by significantly increasing the expression of SIgA and decreasing the population of Tregs in SAP rats.

### Analysis of the Chemical Profile of FTRAs

**FIGURE 5:**
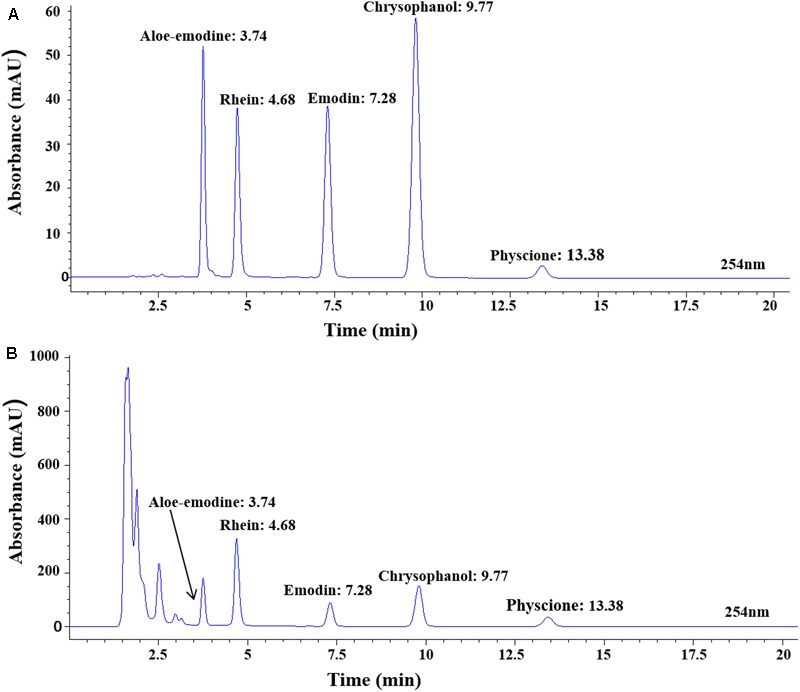
The chemical profile of FTRAs determined at Absorbance of 254 nm optical density (OD) by HPLC-UV analysis. **(A)** The standard curves for each compound. **(B)** The chemical contents of FTRAs: aloe-emodine, rhein, emodin, chrysophanol and physcione and their retention times at 3.74, 4.68, 7.28, 9.77, and 13.38 min, respectively.

To determine the chemical profile of FTRAs, we study its chemical fingerprint at 254 nm optical density (OD) by HPLC-UV analysis and the data are shown in **Figure 5**. The analysis revealed that FTRAs consist of five compounds on their UV spectra including aloe-emodine, rhein, emodin, chrysophanol, and physcione and their retention times are 3.74, 4.68, 7.28, 9.77, and 13.38 min, respectively.

## Discussion

Severe acute pancreatitis has relatively high morbidity and mortality and lacks effective treatment (Myer et al., 2013; Cen et al., 2016). The intestinal immune system plays an important role but the pathogenesis has not been fully clarified in SAP (Zhang et al., 2008). The normal intestinal mucosal barrier maintains a dynamic balance and effectively prevents intestinal bacteria and ET translocation (Garside et al., 2004). The impairment of intestinal immunity and barrier function may lead to the increase of the permeability of the intestinal mucosa barrier to affect intestinal function for effectively preventing bacteria and their products such as ET into the lymphatic vessels of intestines and blood flow, further promoting the release of inflammatory mediators to cause systemic inflammation (Li et al., 2016; Vindigni et al., 2016). In the present studies, we showed that STC induced SAP produced severely pathological injury in the intestinal mucosas and tissues in the rats (**Figure 1A**). At the same time, the blood levels of ET and inflammatory factors such as IL-1β, TNF-α, and NO were significantly increased (**Figure 1B**). The results demonstrated that the intestinal barrier function was severe damaged or destroyed and cannot effectively prevent the translocation of bacteria and ET into the blood in SAP rats. These findings are consitent with the previous reports (Feng et al., 2012; Kang et al., 2017). However, FTRAs significantly reduced intestinal damage and the levels of ET, IL-1β, TNF-α, and NO induced by STC in SAP rats (**Figures 1**). Studies have shown that Rhubarb could regulate intestinal immune function and FTRAs have similar or even more effectiveness in protection of intestinal barrier injury and regulation of intestinal immune function with the advantage of simple composition and smaller doses for treatment (Zhang et al., 2013, 2015, 2016). Our results show that FTRAs are effective in protection of intestinal injury and significantly reduce the levels of ET, NO, and inflammatory factors TNF-α and IL-1β in the blood and/or intestines of SAP rats (**Figure 1**), indicating that the effects of FTRAs on the reductions of ET, NO, and inflammatory factors TNF-α and IL-1β may be related to the recovery of intestinal immune function to further prevent intestinal injury.

Microvascular permeability and tissue edema are the important pathophysiological characteristics of intestinal dysfunction (Woodruff et al., 2016). Determination of tissue EB which is closely attached to albumin reflects inflammatory tissue injury as a method for the examination of the extent of capillary permeability (Diao et al., 2012; Cheng et al., 2016). In addition, the ratio of wet/dry weight is also an important indicator of capillary permeability (Nakagawa et al., 2009). Our results showed that the capillary permeability of intestinal mucosa was significantly increased but FTRAs effectively decreased the elevated capillary permeability in SAP rats by EB content and the ratio of wet/dry weight of intestinal tissues (**Figure 2**), suggesting that FTRAs can obviously decrease intestinal edema and improve intestinal function in SAP rats.

Intestinal immune functions are mainly relied on non-specific and specific immunities for secretions of SIgA and modulation of immune cells in the intestinal mucosal surface (Mantis et al., 2011; Cader and Kaser, 2013). Currently, much attention has been paid to the role of innate immunity in the development of SAP (Hoque, 2016; Shamoon et al., 2016). The innate immune cells in the intestine can activate nuclear factor kappa B (NF-κB) and inflammasome signal pathway through pattern recognition receptors [Toll-like receptors (TLRs) and Nod-like receptors (NLRs)], rapidly initiating inflammatory responses (Hoque et al., 2014). NLRP3 inflammasome is one of the best characterized NLR family members and a key component of the innate immune system (Cassel et al., 2009; Chang, 2013). It is composed of NLRP3, ASC and caspase-1 and an important regulatory factor of inflammation and cell apoptosis (Agostini et al., 2004). NLRP3 inflammasome plays a critical role in activation of caspase-1, release of IL-1β, and pathogenesis of SAP (Hoque et al., 2011; Youm et al., 2011; Lu et al., 2017). ASC activates caspase-1 precursors into active form and caspaes-1 not only plays an important role in signal transduction but also in activation of inflammasome in inflammatory response (Mogensen, 2009). Caspase-1 is able to transform in active pro-IL-1β and pro-IL-18 to biological active IL-1β and IL-18 (Hitzler et al., 2012). IL-1β is a major inflammatory factor and it binds to its receptor after released into extracellular space to induce inflammatory mediators and cause inflammation (Hoque et al., 2011; Youm et al., 2011). The present studies demonstrated that the expressions of NLRP3, ASC and casepase-1 in small intestines and MLN cells were significantly higher in SAP rats than that of normal rats. Interestingly, FTRAs significantly decreased the expressions of NLRP3, ASC and casepase-1 in small intestine and MLN cells in SAP rats (**Figure 3**). Our previous studies have shown that FTRAs could inhibit the expression of NF-kB (Yang et al., 2007). However, this is the first time to show that FTRAs inhibited the release of IL-1β by reducing the expressions of NLRP3 inflammasome, ASC and casepase-1 to further alleviate excessive innate immune mediated inflammatory response and intestinal injury in SAP.

Secretary immunoglobulin A is a main immunoglobulin protein of the intestinal mucosal surface secreted by B lymphocytes after differentiating into plasma cells via antigen stimulation and activation; its expression is inversely proportional to the severity of SAP (Sun et al., 2013). Intestinal mucosal surface immunoglobulin SIgA is the first line of intestinal mucosal defense and plays a key role in humoral immunity (Diebel et al., 2003; D’Auria et al., 2013). Wang et al. (2007) found that the levels of SIgA in the peripheral blood were significantly decreased in SAP rats. Clinical study also found that the levels of SIgA were significantly declined in the peripheral blood of patients with SAP (Sun et al., 2013). In our results, the levels of SIgA in the intestines of SAP rats were also significantly decreased compared to that of normal rats, indicating that the function of B lymphocyte of SAP rats was severely impaired and resulted in significant reduction of SIgA secretion. The results are consistent with the results from previous studies (Wang et al., 2007; Sun et al., 2013). Interestingly, FTRAs significantly (*p* < 0.01) increased the expression of SIgA in the small intestinal tissues in SAP rats (**Figure 4A**). Our data demonstrated that FTRAs could promote the secretion of SIgA to reduce bacteria and ET into the blood and intestinal damage in SAP rats (**Figures 1, 4A**). Therefore, the effect of FTRAs on the protection of intestinal epithelial cells from injury may be, at least in part, through the upregulation of the levels of SIgA in the small intestinal tissues and, thereby regulating the specific immune response in SAP rats.

The cells play a central role in the body’s specific immune response. Th1 cells induce cellular immunity mediated by inflammatory cytokine, while Th2 cells are associated with humoral immune responses (Mosmann and Coffman, 1989; Schmidt et al., 2017). Studies have shown that Th1 cells secreted excessive amounts of inflammatory cytokines such as TNF-α, IFN-γ, and IL-1β, while Th2 cells mainly induced humoral immunity mediated by anti-inflammatory cytokines in patients with early SAP (Mosmann and Coffman, 1989; Oiva et al., 2010). IFN-γ secreted by Th1 cells can induce apoptosis in intestinal epithelial cells and activates macrophages to further secrete TNF-α (Duque and Descoteaux, 2014). Thus, TNF-α is a vital factor to connect innate immunity and adaptive immune response and plays a key role in the pathogenesis of SAP. Th1 cells inhibits Th2 cells after their activation by antigens to induce Th1/Th2 cytokine dynamic imbalance and result in a series of inflammatory reaction and aggravating intestinal injury, further triggering the productions of systemic inflammatory response syndrome and other serious consequences in patients with SAP (Rangel-Frausto et al., 1995). The balance of Th1/Th2 is crucial for maintaining homeostasis in the body, the change in the ratio of Th1/Th2 has a profound impact on the body immune function and imbalance of Th1/Th2 can cause SAP (Sattler et al., 2009; Hussein and Ahmed, 2016). Therefore, reduction of the ratio of Th1/Th2 to restore their balance may be an effective strategy to prevent further deterioration in SAP patients. In the present results, our results showed that the ratio of Th1/Th2 was dramatically increased by increasing Th1 and decreasing Th2 to induce excessive inflammatory response with high levels of TNF-α and IL-1ß in SAP rats (**Figures 1, 4B**). Interestingly, FTRAs significantly decreased the elevated ratio of Th1/Th2 and restored their balance to near normal level with significant lower levels of TNF-α and IL-1β in SAP rats (**Figures 1, 4B**), suggesting that FTRAs may be effective in regulation of intestinal adaptive immunity.

Tregs are suppressive T cells that have an essential role in maintaining the balance between immune activation and tolerance (Hart et al., 2015; Hussein and Ahmed, 2016). The transcription factor CD4 + Foxp3 of Tregs is identified as the most specific markers of Tregs (Hori et al., 2003; Yun et al., 2015). Tregs exert their immunosuppressive effects by inhibiting INF-γ secretion and promoting IL-4 secretion (Jin et al., 2013). In normal condition, IL-4 secreted by Th2 cells can prevent the activation of macrophages and production of TNF-α and IL-1β to inhibit inflammation and reduce tissue damage (Hart et al., 2015; Hussein and Ahmed, 2016). Studies have shown that Tregs were decreased and negatively correlated with serum concentration of TNF-α and the decrease of Tregs may be responsible for uncontrolled immune activation to cause excessive inflammatory response in SAP (Ding et al., 2007; Pan et al., 2017). However, another study has showed that the Tregs are abnormal high in SAP patients and elevated circulating Tregs is an independent prognostic biomarker for patients with ASP (Minkov et al., 2017). In the present study, we found that the levels of CD4 + Foxp3 and Tregs population were significantly increased with positive correlation with the blood levels of ET, TNF-α, and IL-1β in SAP rats. The finding is inconsistent with some studies (Ding et al., 2007; Pan et al., 2017), but is supported by others (Xiong et al., 2012; Minkov et al., 2017). Our results indicated that the Tregs were activated under inflammatory condition and intestinal mucosal immunity was shifted from an over-inflammatory state to an immunosuppressed state in ASP rats. The possible reason for the different finding from different studies may be related to observation time difference, the immune response was varied at different times so multiple time points observation may be necessary to study the dynamic change of immune function in SAP to help better understand of the immune response at different stages. In addition, different studied samples may be another possible reason. We used MLN cells for the present study while other groups used blood sample for their studies. Interestingly, FTRAs can significantly decrease the expression of Foxp3 and population of Treg (**Figure 4C**). The present results showed that FTRAs are effective in regulation of the intestinal immune function in SAP rats by inhibition of NLRP3 inflammasome, upregulation of SIgA, restoration of Th1/Th2 cells balance, and decrease of Tregs number. Previous study has shown that the main ingredients of FTRAs include rhubarb, emodin, aloe-emodin and their glycosides (Aichner and Ganzera, 2015). In the present study, we revealed that FTRAs consist of five compounds including aloe-emodine, rhein, emodin, chrysophanol, and physcione by UHPLC-UV analysis (**Figure 5**). We will investigate the effects of individual compound on intestinal protection and immune modulation in animal models to identify the most effective compound(s) for the treatment of SAP in the future study.

## Conclusion

Our studies elucidated that SAP induced by STC could destroy intestinal immune functions to trigger specific manifestations including intestinal injury, increase of ET, TNF-α, IL-1β, NO, and intestinal permeability, activation of NLRP3 inflammasome, increase of Tregs number, and induction of Th1/Th2 imbalance in rats. However, FTRAs protected the intestinal injury and improved intestinal mucosal function through decrease of ET, TNF-α, IL-1β, NO and intestinal permeability and downregulation of the expression of NLRP3 inflammasome to modulate intestinal non-specific immune response. Furthermore, FTRAs also increased the expression of SIgA, decreased the number of Tregs, and restored the balance of Th1/Th2 to modulate intestinal specific immune response in SAP rats. Therefore, FTRAs may have the potential to be developed as the novel agent for the treatment of SAP clinically. However, further studies are required to find out the active ingredients of FTRAs to identify the purified compound(s) to be developed and understand the exact mechanism of action, particularly the intestinal immune system including the role of FTRAs in the TLRs and NLRs signaling pathways, associated with their efficacy in SAP treatment.

## Author Contributions

All authors listed have made contribution to the work and approved it for publication. YX and SC conceived and designed the experiments. LF, LC, LW, YZ, and YX performed the experiments. LC, LF, LW, and SC analyzed the data. DQ, QS, and JW contributed reagents/materials. LF, LC, LW, YZ, YX, and SC wrote the paper.

## Conflict of Interest Statement

The authors declare that the research was conducted in the absence of any commercial or financial relationships that could be construed as a potential conflict of interest.
